# Historisches zur Ethik in der Psychiatrie: Vom Ende der Humanität zur Menschenrechtskonvention

**DOI:** 10.1007/s00115-024-01658-w

**Published:** 2024-05-27

**Authors:** Heinz-Peter Schmiedebach

**Affiliations:** Institut für Geschichte und Ethik der Medizin, Universitätsklinikum Hamburg-Eppendorf, Martinstraße 52, 20246 Hamburg, Deutschland

**Keywords:** „Kriegsneurotiker“, „Hungersterben“, Krankenmorde, Psychiatriereform, UN-Behindertenrechtskonvention, “War neurotics”, Death by starvation, Murder of invalids, Psychiatry reform, United Nations Convention on the Rights of Persons with Disabilities

## Abstract

Um 1900 war die Etablierung der akademischen Psychiatrie vollzogen. Gleichzeitig verschob sich das professionelle Selbstverständnis der Psychiater angesichts der gesellschaftlichen Krisenphänomene hin zu einem Selbstbild, nach dem die Psychiatrie ihre Expertise in den Dienst an Volk und Vaterland zu stellen hatte. Dies kam besonders im Ersten Weltkrieg im brutalen Umgang mit den sog. Kriegsneurotikern zum Ausdruck. Im Zusammenhang mit dem sog. Hungersterben von ca. 70.000 Anstaltsinsassen erörterte Karl Bonhoeffer in der Nachkriegszeit eine Wandlung des Humanitätsbegriffes. Als schlimmste Konsequenz der Abkehr vom Humanitätsdenken sind die im Nationalsozialismus erfolgten Morde an den Kranken zu nennen. Legitimierungen solcher Verbrechen mit Hinweisen auf eine Kollektivethik, wie es Karl Brandt versuchte, erscheinen jedoch wenig überzeugend. Die in den 1960er-Jahren initiierten Psychiatriereformen und seit 2008 in Kraft getretene Behindertenrechtskonvention der Vereinten Nationen haben Voraussetzungen für eine unterstützende und zwangsreduzierte Psychiatrie geschaffen, wobei jedoch noch viele Fragen, auch im Rechts- und Sozialsystem, zu klären sind.

## Abstract (en)

The establishment of academic psychiatry was completed around 1900. Simultaneously, in view of the societal crisis phenomenon the professional self-concept of the psychiatrist was shifted to a self-image, according to which psychiatry had to place its expertise at the service of the people and the country. This was particularly expressed in World War I in the brutal dealing with the so-called war neurotics. In association with the so-called death by starvation of ca. 70,000 institution inmates, in the post-war period Karl Bonhoeffer debated a transformation of the term humanitarianism. The worst consequence of the rejection of humanitarian thoughts are the murders of invalids under National Socialism; however, legitimization of such crimes by alluding to collective ethics, as attempted by Karl Brandt, seems to be less than convincing. The reform of psychiatry initiated in the 1960s and the United Nations Convention on the Rights of Persons with Disabilities, which came into force in 2008, have achieved prerequisites for a supportive psychiatry with reduced coercion, whereby many questions also in the legal and social systems must still be clarified.

## „Kriegszitterer“ und Hungersterben im Ersten Weltkrieg

Um 1900 war die Etablierung der akademischen Psychiatrie in Deutschland vollendet, gleichzeitig waren vielerorts in einer dritten Gründungswelle weitere psychiatrische Großanstalten entstanden, die sich schnell füllten und bald überfüllt waren. Die klassische Moderne ab ca. 1880 war durch zahlreiche widersprüchlich verlaufende Entwicklungen gekennzeichnet. Die Neuverteilung sozialer und politischer Entscheidungskompetenzen führte in mehrerlei Hinsicht zu Verwerfungen und Auseinandersetzungen. Diese riefen ein Krisenempfinden hervor, das zudem durch neue Kommunikationstechnologien, umfassende Beschleunigungen und Dissoziationsphänomene bei vielen Menschen Irritationen, Umstellungsschwierigkeiten und Erschöpfungszeichen auslöste; die Neurasthenie war ein Ausdruck dieser Umstände. Die zunehmenden antimodernen und kulturpessimistischen Sichtweisen, die durch die Degenerationslehre verstärkt wurden, verschoben auch das professionelle Selbstverständnis und veränderten die Sichtweise auf die Kranken. Viele Psychiater sahen sich angesichts der gesellschaftlichen Krisenphänomene aufgerufen, ihre Expertise in den Dienst von Volk und Vaterland zu stellen. Dabei änderte sich auch die Sicht auf die Kranken. Sie wurden mehr und mehr zu einer Gruppe, die das Wohl des Staates, der Nation, der „Rasse“ auf vielfältige Weisen zu gefährden schien. Die viel beschworene Humanität im Umgang mit den Kranken, die bis in die 70er-Jahre des 19. Jahrhunderts noch als deutliche Referenz in der Diskussion vorhanden war, spielte nunmehr keine Rolle, ja wurde sogar in den 1920er-Jahren mit dem pejorativen Begriff der „Humanitätsduselei“ als geradezu gefährlich naive und schädliche Anwandlung abgewertet.

Dieser Habitus, der den Psychiater als Kämpfer für das Wohl von Volk und Vaterland auswies und der auch die Interessen der Patientinnen und Patienten diesem Wohl unterordnete, erhielt während des Ersten Weltkrieges nochmals einen deutlichen Schub. Dies ist am Umgang mit den sog. Kriegsneurotikern und mit dem sog. Hungersterben [14, S. 83–107] und den psychiatrischen Reaktionen darauf abzulesen. Zeitgenossen wie Sigmund Freud und Karl Bonhoeffer konstatierten in diesem Zusammenhang eine Veränderung der Humanität. Spätestens ab 1916 war bei den mit den sog. Kriegsneurotikern befassten Psychiatern die allergrößte Mehrheit der Auffassung, dass die Psychiatrie bei diesen Personen „falsche“ Vorstellungen, einen „schwachen Willen“ oder einen „ethischen Defekt“ bekämpfen oder umpolen müsse [14, S. 118]. In praxi führte dies zu einem Vorgehen, das auf durch Schmerzzufügung hervorgerufene „Schocks“, Suggestion und Hypnose setzte und die militärische Hierarchie und Disziplin in die Behandlungsstrategie integrierte. Mit dieser Behandlung sollte eine möglichst schnelle Wiederherstellung der Funktionsfähigkeit des Soldaten erreicht werden. Dem Kranken wurde nun gewissermaßen der Befehl erteilt zu gesunden, der Erfolg der Behandlung sei vom „Siegeswillen“ des Arztes abhängig. Die Metapher des militärischen Kampfes dominierte das Denken. Der kriegsneurotische Patient, der wegen seiner Symptome nicht mehr kämpfen konnte, die Kampfkraft schwächte und so von innen das Geschäft des Feindes betrieb, wurde selbst mit dem Feind gleichgesetzt. Im Arzt-Patient-Verhältnis fanden nun nicht mehr Hilfesuchender und Helfer zueinander, sondern Gegner. Auf Seiten der Ärzte sei der unbedingte Siegeswille nötig, um die moralischen und willentlichen Defizite der „Kriegsneurotiker“ erfolgreich zu bekämpfen und abzustellen. Im Gegensatz zu der modernen Material- und Maschinenschlacht auf dem Feld kam es in den psychiatrischen Lazaretten zum Kampf Mann gegen Mann [14, S. 131]. Sigmund Freud äußerte sich nach dem Krieg zu dem von betroffenen Soldaten und politischen Verbänden heftig kritisierten Verhalten der Psychiater. Er war sich des medizinethischen Dilemmas bewusst.

„Der unlösbare Konflikt zwischen den Anforderungen der Humanität, die sonst für den Arzt maßgebend sind, und denen des Volkskrieges mußte auch die Tätigkeit des Arztes verwirren“ (zit. n. [5, S. 33]).

Letztlich umging Freud die Lösung des Konflikts. Die unbedingte Einbindung der Medizin in die militärischen Abläufe und Ziele schätzte er zwar als konfliktträchtig ein, die daraus resultierenden Probleme aber als unlösbar. Der Arzt hatte sich den Erfordernissen des Krieges unterzuordnen, sollte aber seine Macht gegenüber den psychiatrischen Lazarettpatienten nicht brutal zum Ausdruck bringen.

Zum „Hungersterben“ von mehr als 70.000 Patientinnen und Patienten in deutschen Anstalten während des Ersten Weltkriegs äußerten sich nach dem Krieg Emil Kraepelin und Karl Bonhoeffer. Kraepelin sprach von einer Erleichterung der „wirtschaftlichen Last, die unheilbare Geisteskranke bedeuten“, rechnete jedoch damit, „daß sich die Folgen der Hungerblockade noch lange Zeit hindurch in einer geringeren Widerstandsfähigkeit unserer Bevölkerung geltend machen werden“ [7]. Er wog also die Reduktion der sogenannten wirtschaftlichen Last durch das Sterben der Unheilbaren gegen Abnahme der allgemeinen Widerstandfähigkeit der Bevölkerung ab. Während er das Verhungern der Unheilbaren als ökonomisch vorteilhaft betrachtete, war für ihn das Hungerleiden der Bevölkerung wegen der gesundheitsschädlichen Folgen jedoch von großem Nachteil.

Bonhoeffers Abwägungen zum Hungersterben in den Anstalten fielen anders aus:

„Fast könnte es scheinen, als ob wir in einer Zeit der Wandlung des Humanitätsbegriffes stünden … und dass wir in den Hungerjahren des Krieges uns damit abfinden mussten, zuzusehen, dass unsere Kranken in den Anstalten in Massen an Unterernährung dahinstarben, und dies fast gutzuheißen in dem Gedanken, dass durch diese Opfer vielleicht Gesunden das Leben erhalten bleiben konnte“ [2].

Ebenso wie Freud erkannte auch Bonhoeffer ein Ende der Humanität durch den Krieg, aber auch er griff zu einer Abwägung, einer konjunktivischen Überlegung, indem er ein Opfer annahm, durch welches „vielleicht“ Gesunden das Leben hätte erhalten werden können. Dieses von Bonhoeffer angesprochene psychiatrische Krankenopfer unterstreicht, wie die Asymmetrie zwischen Kranken und Ärzten im Ersten Weltkrieg erstmals einen tödlichen Charakter annahm. Im Krieg, dem größten vorstellbaren Schadensereignis per se, wurden alle ärztlich-psychiatrischen Überlegungen zur Schadensreduktion an Patienten und Patientinnen, zur Selbstbestimmung der Kranken nicht nur relativiert, sondern aufgehoben. In dieser Lage, in der das Überleben des Volkes und der Nation von modernen Optimierungs- und Mobilisierungsstrategien aller menschlichen Ressourcen abzuhängen schien, wurde Menschwürde auch im Kontext psychiatrischer Versorgung zum Luxusrelikt aus romantischen Zeiten.

## Krankenmorde und „kollektivethische“ Rechtfertigung?

Geprägt von diesen durch den Krieg verstärkten Sichtweisen publizierten der Jurist Karl Binding und der Psychiater Alfred Hoche im Jahre 1920 ihr Buch über die Vernichtung lebensunwerten Lebens. Explizit wurden die Vernichtungsgedanken damit begründet, dass sich Deutschland auf einer schwierigen Expedition befinde, „bei welcher die größtmögliche Leistungsfähigkeit Aller die unerläßliche Voraussetzung für das Gelingen der Unternehmung bedeutet, und bei der kein Platz ist für halbe, Viertels- und Achtels-Kräfte“ sei [1, S. 55]. Schließlich wurden im nationalsozialistischen Staat über 300.000 Menschen ermordet unter maßgeblichem Mitwirken von Ärzten und Ärztinnen. Angesichts mancher Verteidigungsversuche der involvierten ärztlichen Personen stellt sich die Frage, was davon zu halten ist, dass manche der an den Ermordungen Beteiligten sich mit Mitleid und ethischen Überlegungen zu rechtfertigen versuchten? Wie ist der Hinweis auf eine Kollektivethik, die das Gemeinwohl zur Priorität erklärte, zu bewerten?

Karl Brandt, im Nürnberger Ärzteprozess zum Tode verurteilt und maßgeblicher Organisator der Krankenmorde [13, 20], führte aus, dass er vom menschlichen Empfinden geleitet worden sei und nie etwas anderes beabsichtigt habe, als den armseligen Wesen das qualvolle Dasein abzukürzen [9, S. 208]. Bezüglich der Menschenversuche meinte er, im Krieg müsse die einzige ethische Entscheidungsgrundlage die Relevanz des Versuches für das „Volkswohl“ und die Erhaltung der Kampfkraft sein. Kurz, die Humanexperimente hätten dazu beigetragen, das Leben der eigenen Soldaten zu retten:

„Der Sinn ist das Motiv, das der Gemeinschaft gilt“ (zit. n. [4, S. 349]).

Hierzu ist festzustellen, dass die 1931 vom Reichsgesundheitsrat erlassenen Richtlinien für neuartige Heilbehandlungen und die Vornahme wissenschaftlicher Versuche, die eine Ausweitung der Schutzrechte der Probanden und Probandinnen im Sinne eines „informed consent“ bedeuteten und unter Punkt 7 auch die Ausnutzung einer sozialen Notlage bei der Erprobung neuartiger Heilmittel untersagten, nie in der NS-Zeit ausgesetzt oder revidiert wurden. Im Gegenteil waren sie bis 1942 mehrmals in mehreren Auflagen eines Buches, das für die Ärzteschaft relevante Gesetze und Verordnungen publizierte, abgedruckt [12, 15]. Somit ist klar, dass es zu keiner offiziellen Revision der Regeln für Menschenversuche während der NS-Zeit kam und die bereits 1931 aufgestellten Richtlinien auch in den späteren Jahren öffentlich leicht zugänglich waren.

Hinsichtlich der von Brandt benutzten Mitleids- und Erlösungsrhetorik im Kontext der Krankenmorde gilt es zu bedenken, dass die Tötungen der psychisch Kranken erst ein Anfang waren, gewissermaßen eine Testphase, um das millionenfache Töten auch von anderen als „minderwertig“ eingeschätzten Menschen zu erproben. Es ging also um Millionen anderer Menschen, die mit weitreichenden Mordaktionen ausgelöscht werden sollten. Explizit antwortete Brandt im Nürnberger Ärzteprozess auf die entsprechenden Fragen, dass er durch „Ausrottung der Juden“ und der KZ-Häftlinge nicht belastet sei [9, S. 209]. Das kollektivethisch hergeleitete Wohl der Gemeinschaft, das an sich harmlos klingende Ziel des „Volkswohls“, auf das sich Brandt berief, war aber untrennbar mit dem millionenfachen Mord von als „minderwertig“ betrachteten Menschen und Volksgruppen verknüpft, was Brandt natürlich wusste. Insofern bedeutet die von ihm vorgenommene Unterscheidung der gesamten Gruppe der Ermordeten in eine Klasse, die er als armselige Wesen aus Mitleid erlösen wollte, und in eine andere, deren Ermordung er im Interesse des vermeintlichen „Volkswohl“ als notwendig ansah und die ihn nicht belastete, ein besonders problematischer Versuch der Rechtfertigung der Ermordung der Kranken, da die Täter die Ermordung der verschiedenen Gruppierungen als ein Zusammenhängendes sahen, in diesem Sinne planten und im Interesse eines vermeintlichen „Volkswohls“ auch sukzessive durchführten.

## Psychiatriekritik, Reformen und Menschrechtskonvention

In den 1960er-Jahren entstand eine internationale Diskussion, eingebettet in verschiedenartige Emanzipationsbewegungen, in der es auch um die Freiheit der psychisch Kranken ging. In Deutschland war die menschenunwürdige Unterbringung psychisch Kranker in der Öffentlichkeit Thema. Das ZDF benannte 1970 im „Gesundheitsmagazin Praxis“ die Zustände in der Psychiatrie als „Gesundheitsnotstand Nr. 1“ [19]. Die sich international entfaltende Diskussion stellte dabei auch die grundsätzliche Frage, was eine psychische Krankheit sei, ob es diese überhaupt gebe, welche Therapien in Anwendung kommen, wie die Forschung betrieben und wie die Unterbringung gestaltet sein müsse, einschließlich der Forderung, psychiatrische Großkliniken aufzulösen und die professionelle Psychiatrie zu entmachten. Dies war die Zeit, in der eine „Antipsychiatrie“ ihre höchst unterschiedlichen Stimmen erhob und in der sich eine neue Sozialpsychiatrie etablierte, wobei zunehmend auch verschiedene Berufe aus dem Sozialbereich in eine sich immer weiter differenzierende und fragmentierende psychiatrische Versorgung eingebunden wurden. Zudem war vom Weltärztebund in einer weitreichenden ethischen Debatte zu Forschungsfragen die Deklaration von Helsinki 1964 verabschiedet worden. All diese Phänomene sind Ausdruck eines umfassenden in Gesellschaft und Politik erkennbaren Veränderungs- und Reformklimas. Nicht zuletzt war die politische Bereitschaft, mit einer Psychiatrieenquete eine ausführliche Bestandsaufnahme der Psychiatrie in Westdeutschland vorzunehmen und Reformen vorzutragen, auch ein Produkt dieser gesellschaftlichen Stimmung [6, 16].

In den letzten 70 Jahren gab es in den westlichen modernen Gesellschaften aber auch zunehmende Säkularisierungsphänomene, die traditionelle normative Instanzen, wie z. B. die christlichen Kirchen, in den Hintergrund drängten. In dieser sich neu entfaltenden Wertevielfalt sollten Erörterungen medizinethischer Inhalte einen neuen Konsensprozess in Gang setzen, um zumindest einige für die Ärzteschaft allgemeingültige Regeln zu formulieren. Spätestens seit den 1980er-Jahren waren grundlegende Prinzipien, wie Respekt vor der Selbstbestimmung, das Nichtschadensgebot und das Handeln zum Wohle des Kranken wie auch Gerechtigkeit akzeptiert. Doch diese Prinzipien waren bestenfalls Leitplanken. Wie diese im Einzelfall umzusetzen waren, bedurfte weiterer Überlegungen. Um hierbei Hilfestellung zu gewähren, griff man zur Institutionalisierung. Man schuf Ethikkommissionen, welche die medizinische Forschung unter ethischen Gesichtspunkten begleiten sollten, und richtete klinische Ethikkonsile ein, die sich ethischen Fragen im klinischen Alltag widmen sollten [18]. Die sich immer weiter differenzierende ethische Debatte hat in den letzten 70 Jahren zu Tausenden von Publikationen, zur Gründung zahlreicher Journale, zur Etablierung von Fachgesellschaften geführt, die sich ausschließlich mit diesen Fragen befassen, die allerdings im Rahmen dieses Beitrags nicht ansatzweise vorgestellt werden können.

Das am 03.05.2008 in Kraft getretene und seit dem 26.03.2009 für Deutschland verbindliche Übereinkommen der Vereinten Nationen über die Rechte von Menschen mit Behinderungen ist ebenfalls ein politisches Ergebnis dieser internationalen Debatten [3]. Dieses universelle Vertragswerk möchte den vollen und gleichberechtigten Genuss aller Menschenrechte und Grundfreiheiten bei allen Menschen mit Behinderungen gewährleisten und die Achtung der ihnen innewohnenden Würde fördern. Zu den Menschen mit Behinderungen zählen Menschen, die langfristige körperliche, seelische, geistige oder Sinnesbeeinträchtigungen haben. Konkret soll diesen Personen in vollkommener Gleichberechtigung jeglicher Zugang zu allen gesellschaftlichen Institutionen und Rechten gewährleistet werden, wie z. B. zur Bildung, Regelung eigener finanzieller Angelegenheiten, Wahl des Wohnungsortes, Recht auf Nachwuchs etc. Die Menschenrechte werden in Tiefe und Reichweite deutlich ausgeweitet. Verschiedene Rechte ziehen eindeutig die Grenzen einer Zulässigkeit von Zwang sehr viel enger als zuvor. Ob die Konvention einen vollständigen Ausschluss von Zwangsmaßnahmen vorsieht, ist jedoch umstritten. In Anbetracht einer durch die Nichtbehandlung gegebenen Selbst- oder Fremdgefährdung oder sozialen Verwahrlosung besteht deswegen die Gefahr, unerwünschte und humanitär fragwürdige Wirkungen hervorzurufen. Zu den Regelungen der Konvention gehören z. B. das Recht auf Freiheit und Sicherheit (Art. 14), nach dem kein Freiheitsentzug erlaubt ist, sodann das Recht auf Freiheit von Folter, grausame, erniedrigende oder unmenschliche Behandlung (Art. 15), nach dem medizinische Zwangsbehandlung, Isolation und Fixierung infrage gestellt werden. Schließlich ist noch das Recht auf rechtliche Handlungsfreiheit (Art. 12, Abs. 1 u. 2) zu nennen, wonach eine Fremdbestimmung ausgeschlossen ist. Diese Regelungen betreffen ganz besonders die Zwangsmaßnahmen und Zwangsmedikation in der Psychiatrie. Zwangsmaßnahmen und Zwangsmedikation sind nach wie vor verbreitet, wenn auch nur unter erschwerten Bedingungen möglich. Dennoch nehmen sie zu, wie verschiedentlich aufgezeigt [10, S. 1222]. Ob dies einer erhöhten Aufmerksamkeit und Meldung geschuldet ist oder eine tatsächliche Zunahme bedeutet, sei dahingestellt. Diese Maßnahmen betreffen aber in Deutschland Tausende von Personen im Jahr und werden von den Betroffenen häufig als erniedrigend oder als traumatisierend empfunden.

Nun kann man fragen, ob nicht eine Gesellschaft hier in die Verantwortung zu nehmen ist und dafür zu sorgen hat, dass die Rechte der UN-Behindertenkonvention, die immerhin seit 14 Jahren in Deutschland Gültigkeit besitzt, umgesetzt werden. Wenn wir dies bejahen, dann muss die Gesellschaft durch die Bereitstellung von Kapazitäten und Kompetenzen dies auch tun. Wenn auch Gesetzesänderungen erste Schritte in dieser Hinsicht bedeuten können, so ist aber zudem die Psychiatrie aufgefordert, gemeinsam mit Betroffenen und Angehörigen nach grundsätzlichen Strukturveränderungen, Veränderung von Einstellungen beim Personal im Sinne weg von einer fürsorglich-patriarchalischen hin zu einer menschenrechtsorientierten und unterstützenden Perspektive zu suchen. Zu diesen Fragen und nötigen Veränderungen sind schon zahlreiche grundlegende Überlegungen publiziert worden, in denen z. B. die Vermischung von ordnungspolitischen und medizinischen Funktionen der Psychiatrie aufgelöst werden sollen [17, 21]. Auch liegen Publikationen zu konkreten Erfahrungen psychiatrischer Institutionen mit offenen Türen der Stationen, Reduzierung der Bettenzahl, Einrichtung von Rückzugsmöglichkeiten, Deeskalisierungsstrategien etc. vor [8]. Doch sind noch viele Fragen offen. Zudem verlangt eine Psychiatrie ohne Zwang nicht nur eine grundlegende Erörterung der gegenwärtigen inhaltlichen und strukturellen Grundlagen der Psychiatrie, sondern würde in Anbetracht einer starken Medikalisierung des Rechts- und Sozialsystems auch eine Reform in diesen beiden Sektoren nötig machen, wie dies Dirk Richter aufzeigt [11]. So sind noch manche Aspekte einer Psychiatrie ohne Zwang in ihren weit über das Fach Psychiatrie hinausreichenden Dimensionen keineswegs hinreichend erfasst und schon gar nicht in Öffentlichkeit und Politik mit der nötigen Tiefe und Konsequenz erörtert, geschweige denn zu einem wirkungsvollen gesundheitspolitischen Programm ausformuliert. Es scheint deshalb sinnvoll, zunächst eine umfassende Bestandsanalyse zum Zwang in der Psychiatrie in Deutschland zu erarbeiten, eine Ursachenforschung zu betreiben und schließlich Vorschläge zu weitreichenden Strukturveränderungen auf all den infrage stehenden Gebieten zu machen. In einem weiteren Schritt könnten die Ergebnisse dieser Analysen und Debatten in einem psychiatrischen und sozialpolitischen Reformprogramm gebündelt werden, um auf dieser Grundlage in einer breiten Öffentlichkeit und in der Politik, rund 50 Jahre nach der Psychiatrie-Enquete, diese Ideen zu erörtern und umzusetzen.

## Fazit für die Praxis

Die historische Analyse psychiatrischer Entwicklungen in ihrer Verwobenheit mit gesellschaftlichen Faktoren kann die Sichtweise auf Faktoren, die eine aktuelle psychiatrische Praxis bestimmen, sensibilisieren und das Spektrum für mögliche Optionen innovativer psychiatrischer Versorgung erweitern.
